# Amplicon Sequencing as a Potential Surveillance Tool for Complexity of Infection and Drug Resistance Markers in *Plasmodium falciparum* Asymptomatic Infections^[Author-notes jiac144-FM3]^

**DOI:** 10.1093/infdis/jiac144

**Published:** 2022-04-16

**Authors:** Kevin Wamae, Kelvin M Kimenyi, Victor Osoti, Zaydah R de Laurent, Leonard Ndwiga, Oksana Kharabora, Nicholas J Hathaway, Jeffrey A Bailey, Jonathan J Juliano, Philip Bejon, Lynette Isabella Ochola-Oyier

**Affiliations:** Kenya Medical Research Institute (KEMRI)–Wellcome Trust Research Programme, Kilifi, Kenya; Kenya Medical Research Institute (KEMRI)–Wellcome Trust Research Programme, Kilifi, Kenya; Centre for Biotechnology and Bioinformatics, University of Nairobi, Nairobi, Kenya; Kenya Medical Research Institute (KEMRI)–Wellcome Trust Research Programme, Kilifi, Kenya; Kenya Medical Research Institute (KEMRI)–Wellcome Trust Research Programme, Kilifi, Kenya; Kenya Medical Research Institute (KEMRI)–Wellcome Trust Research Programme, Kilifi, Kenya; Division of Infectious Diseases, Department of Medicine, School of Medicine, University of North Carolina at Chapel Hill, Chapel Hill, North Carolina, USA; Department of Medicine, University of Massachusetts Medical School, Worcester, Massachusetts, USA; Department of Pathology and Laboratory Medicine, Warren Alpert Medical School, Brown University, Providence, Rhode Island, USA; Division of Infectious Diseases, Department of Medicine, School of Medicine, University of North Carolina at Chapel Hill, Chapel Hill, North Carolina, USA; Department of Epidemiology, Gillings School of Global Public Health, University of North Carolina, Chapel Hill, North Carolina, USA; Curriculum in Genetics and Molecular Biology, School of Medicine, University of North Carolina at Chapel Hill, Chapel Hill, North Carolina, USA; Kenya Medical Research Institute (KEMRI)–Wellcome Trust Research Programme, Kilifi, Kenya; Centre for Tropical Medicine and Global Health, Nuffield Department of Medicine, University of Oxford, Oxford, United Kingdom; Kenya Medical Research Institute (KEMRI)–Wellcome Trust Research Programme, Kilifi, Kenya

**Keywords:** deep sequencing, *ama1*, *mdr1*, genetic diversity, artemisinin-based combination therapy, artemisinin resistance, antimalarial drug resistance

## Abstract

**Background:**

Genotyping *Plasmodium falciparum* subpopulations in malaria infections is an important aspect of malaria molecular epidemiology to understand within-host diversity and the frequency of drug resistance markers.

**Methods:**

We characterized *P. falciparum* genetic diversity in asymptomatic infections and subsequent first febrile infections using amplicon sequencing (AmpSeq) of *ama1* in Coastal Kenya. We also examined temporal changes in haplotype frequencies of *mdr1*, a drug-resistant marker.

**Results:**

We found >60% of the infections were polyclonal (complexity of infection [COI] >1) and there was a reduction in COI over time. Asymptomatic infections had a significantly higher mean COI than febrile infections based on *ama1* sequences (2.7 [95% confidence interval {CI}, 2.65–2.77] vs 2.22 [95% CI, 2.17–2.29], respectively). Moreover, an analysis of 30 paired asymptomatic and first febrile infections revealed that many first febrile infections (91%) were due to the presence of new *ama1* haplotypes. The *mdr1-*YY haplotype, associated with chloroquine and amodiaquine resistance, decreased over time, while the NY (wild type) and the NF (modulates response to lumefantrine) haplotypes increased.

**Conclusions:**

This study emphasizes the utility of AmpSeq in characterizing parasite diversity as it can determine relative proportions of clones and detect minority clones. The usefulness of AmpSeq in antimalarial drug resistance surveillance is also highlighted.

## Teaser

In this study, we highlight the utility of a multiplex amplicon sequencing panel for characterizing parasite diversity in malaria asymptomatic infections. We also emphasize the usefulness of this assay in the surveillance of antimalarial drug resistance.

*Plasmodium falciparum* malaria infections are genetically diverse, and infected hosts may harbor multiple genetically distinct subpopulations of parasites, termed the complexity of infection (COI). These distinct genotypes, also termed clones, result from multiple infectious mosquito bite inoculations or a single mosquito bite inoculation containing several parasite clones [1]. Notably, COI is an important indicator of malaria epidemiology due to its positive association with malaria transmission intensity [2].

The outcomes *of P. falciparum* infections range from severe to mild or uncomplicated febrile episodes to asymptomatic *P. falciparum* infections [3]. Asymptomatic infections are thought to result from partial immunity that controls parasite growth but does not eliminate parasites, as well as from an individual’s ability to tolerate parasites without developing symptoms [4]. In addition to asymptomatic infections fueling onward transmission to mosquitoes [5], they may lead to febrile illness [6]. Using *P. falciparum* length polymorphic markers, some studies have suggested that subsequent febrile episodes may result from genetically similar asymptomatic infections that were detected in the preceding asymptomatic infection [7, 8]. Others have found this to be the result of novel clones [7–13]. These length polymorphic markers mainly rely on gel and capillary electrophoresis that are limited in their ability to discriminate similar-sized alleles [14], resulting in nonspecific amplification [15] and stutter peaks [16]. Consequently, studies may underestimate parasite diversity, are insensitive to low-abundant clones, and do not quantify the relative proportions of circulating clones. Likewise, whole-genome sequencing (WGS) has been used to explore parasite diversity with interpretations based on genome-wide diversity across multiple loci [17]. However, cost is a major barrier to using WGS and remains impractical in many settings because of technical challenges associated with the storage and processing of large-scale data [18].

Amplicon sequencing (AmpSeq), an emerging new tool, is advantageous in that highly polymorphic loci may harbor multiple single-nucleotide polymorphisms in a small region of between 100 and 200 bp. With read depths such as 1000–10 000, AmpSeq has the power to detect minority haplotypes of frequencies as low as 0.1% and at single-nucleotide resolution in experimental mixtures of different parasite haplotypes [19, 20]. Therefore, AmpSeq of a polymerase chain reaction (PCR) product spanning such loci provides high sensitivity and specificity to detect minority clones. Accordingly, the objective of this study was to use AmpSeq to track temporal changes in *P. falciparum* genetic diversity in asymptomatic individuals using both *ama1* and *mdr1* as exemplars of COI and drug resistance, respectively. In addition, a paired analysis of asymptomatic and first febrile infections was analyzed to examine changes in COI.

## METHODS

### Study Design

*Plasmodium falciparum*–positive samples from asymptomatic and first febrile malaria infections were collected from the Junju cohort in Kilifi, a region of moderate to high transmission in coastal Kenya. In this cohort, children are recruited at birth for weekly clinical malaria monitoring until the age of 15 years [21]. There are 2 rainy seasons per year in Kenya during which malaria transmission increases, the long rains from May to July and the short rains in October to November. Since 2007, annual cross-sectional surveys have been conducted in this cohort just before the long rains and we focused on the 2007–2016 timepoints.

From 2007 to 2016, a total of 5403 children were recruited for malaria monitoring. Data from the cross-sectional surveys were used to determine asymptomatic infections while active weekly surveillance data were used to determine febrile malaria episodes. An asymptomatic infection was described as (1) an axillary temperature <37.5°C with no history of fever during the cross-sectional survey; (2) no recent febrile malaria episode within the month before the survey; and (3) no fever within the subsequent 7 days from the date of the survey [6]. A febrile episode was defined based on definitions described for this cohort, that is, ≥2500 parasites/µL by microscopy and an axillary temperature >37.5°C [21]. Accordingly, a total of 1 359 asymptomatic and 419 first febrile infections were identified. We aimed to also include all archived asymptomatic as well as paired asymptomatic and first febrile infections. However, due to the unavailability of paired samples, this was not possible from 2007 to 2008; hence, only asymptomatic samples were included. From 2009 to 2016, paired samples were available and were included. Ethical approval for this study was obtained from the ethics review committee of the Kenya Medical Research Institute (KEMRI) under protocol number SERU 3149. Informed consent was obtained from parents/guardians of all study participants before sample collection.

### *ama1* and *mdr1* Amplification

DNA was extracted from frozen blood using the QIAamp DNA Blood Mini Kit (Qiagen, United Kingdom). Amplicons spanning *ama1* (PF3D7_1133400, nucleotides 441–946) and *mdr1* (PF3D7_0523000, nucleotides 183–719) were generated from each sample (in duplicate) using primers designed in this study (Supplementary Table 1). Each 20-µL PCR assay was set up as follows: 1 µL of template DNA (<25 ng), 0.2 µL of Q5 High-Fidelity DNA Polymerase (0.02 U/µL, New England BioLabs, catalog number M0491L), 1 µL (10 mM) forward primer, each tagged with Roche multiplex identifiers (MIDs, Supplementary Table 1) and 1 µL (10 mM) reverse primer, 0.4 µL (10 mM) dNTPs (New England BioLabs, catalog number N0447L), 4 µL (5X) Q5 reaction buffer (New England BioLabs, catalog number M0491L), and 12.4 µL nuclease-free water. The PCR conditions were as follows: initial denaturation (98°C for 30 seconds), followed by 30 cycles of denaturation (98°C for 10 seconds), annealing (60°C for 30 seconds), extension (72°C for 30 seconds), and final extension (72°C for 2 minutes). PCR products were visualized on 1% (w/v) agarose (Sigma-Aldrich, catalog number A9539-500G) gels stained with RedSafeNucleic Acid Staining Solution (iNtRON Biotechnology DR, catalog number 21141). PCR-negative samples were repeated with 1.5 µL of template DNA.

### Preparation of Control DNA Mixtures for AmpSeq

Hereafter, we use the term “clone” to refer to the entire genetic constitution of a parasite and “haplotype” to mean the set of amino acid polymorphisms found on a single sequence. Genomic DNA extracted from *P. falciparum* reference isolates (3D7 MRA-102G and Dd2 MRA-150G) acquired from the Malaria Research and Reference Reagent Resource Centre (BEI Resources) allowed for the detection of 2 (3D7 and Dd2) *ama1* and 3 (1 from 3D7 and 2 Dd2) *mdr1* haplotypes. Dd2 contained 2 *mdr1* gene copies generated due to adaptation to in vitro culture. Therefore, this yielded the *mdr1*-YY and *mdr1*-FY haplotypes based on the combination of alleles at codons 86 and 184. Six DNA mixtures across 5 dilutions (30 in total) were used to prepare sequencing controls in the following proportions: 100%:100%, 75%:25%, 85%:15%, 95%:5%, and 100%:0% of 3D7 and Dd2, respectively.

### Library Preparation and Sequencing

PCR amplicons were purified using the Zymo ZR-96 DNA Clean and Concentrator-5 Kit (Zymo Research, catalog number D4024) according to the manufacturer’s instructions. The purified products were quantified using Quant-iT dsDNA Assay Kit, High Sensitivity (Thermo Fisher, catalog number Q33120) according to the manufacturer’s instructions. The quantified PCR amplicons were normalized by diluting in EB buffer to achieve equimolar concentrations of 1 ng/µL each across all samples and thereafter mixed to create amplicon pools of nonoverlapping MIDs. Sequence library preparation of the amplicon pools was performed according to the manufacturer’s instructions using the KAPA HyperPrep Kit (Roche, catalog number KK8504) with dual index adapters (Roche, catalog number 08278555702). Successful library preparation was confirmed using a random sample of 20 libraries on the Agilent High Sensitivity D1000 ScreenTape System (Agilent, catalog number 5067-5584). Both the *ama1* and *mdr1* adapter-ligated amplicon libraries were then quantified, normalized to 1 ng each, and mixed to create 1 final pool. Paired-end sequencing (2 × 300 bp chemistry) of the final pool was performed on the Illumina MiSeq platform using the MiSeq Reagent Kit v3 (Illumina, catalog MS-102-3003 number) at the University of North Carolina at Chapel Hill high-throughput sequencing facility.

### Sequence Data Analysis

SeekDeep version 3.0.1 was used for sequence data analysis [20]. First, fastq files were demultiplexed into individual samples based on the MIDs. The paired consensus reads for each sample were trimmed and clustered to estimate the frequency of *ama1* and *mdr1* DNA haplotypes. Haplotypes were discarded if they did not occur in the 2 sample replicates. Moreover, since the lowest ratios of the laboratory sequencing controls included a minor haplotype at 5% frequency (3D7 95% vs Dd2 5%), we also conservatively set a 5% cutoff unless the haplotype was independently detected in additional samples at >5%. Relative frequencies of haplotypes within samples were calculated from the number of reads of each haplotype over the total number of reads per sample. Population haplotype prevalence was calculated using the total number of samples that contained the haplotype over the total number of samples genotyped. COI was defined as the number of unique *ama1* haplotypes in each sample, while *mdr1* haplotypes were generated based on the combination of alleles at codons 86 and 184.

### Statistical Analysis

To estimate the number of samples required to reveal the maximum number of *ama1* haplotypes, rarefaction and extrapolation sampling curves were computed using the iNEXT R package version 2.0.2 [22] with 2000 infections set as the endpoint for extrapolation and at a bootstrap of 10 000. The Student *t* test and analysis variance were used to compare the mean COI between asymptomatic and febrile infections over time. Differences in monoclonal and polyclonal infection proportions were examined using the χ^2^ test. Analysis of COI trends over time was performed using the Mann–Kendall trend test function in the trend package version 1.1.4 in R [23]. All statistical tests were conducted in R version 4.0.2 [24] and all plots were generated using the R packages ggplot2 version 3.3.2 [25] and ggpubr version 0.4.0. Expected *ama1* heterozygosity (*H_e_*) was defined as the probability that 2 randomly selected haplotypes from the population will carry different alleles and was estimated using the following formula, where n was the sample size and Pi was the allele frequency [26]:

$$H_{e}=[n\left(n-1\right)]\left[\left(1-\sum Pi^{2}\right)\right]$$

## RESULTS

### Characteristics of the Study Population

From the initial 1359 asymptomatic and 419 first febrile infections, 535 asymptomatic and 114 febrile malaria microscopy-positive samples were available. The median age of the participants was 8.2 years (range, 0.3–15 years) while the duration of follow-up from the asymptomatic to the first febrile infection varied between 10 and 388 days. Approximately half (47.2% [367]) of the study population was female. The geometric mean parasitemia across all time points was lower in asymptomatic infections (median, 1102 [range, 40–600 482] parasites/µL) compared to febrile infections (geometric mean, 31 186 [range, 40–1 280 001] parasites/µL) (*t* test, *P* < .001). The malaria parasite positivity rate by microscopy in asymptomatic infections fell over time during the annual cross-sectional surveys, but this was not significant (*P* = .13; Table 1).

**Table 1.** Number of Samples Successfully Genotyped From 2007 to 2016

Year	No.	Microscopy Positivity Rate, %	Samples Available (Sequenced %)			
			Asymptomatic Episode		First Febrile Episode	
			*ama1*	*mdr1*	*ama1*	*mdr1*
2007	311	16.2	54 (44.4)	54 (22.2)	0	0
2008	336	30.3	109 (44.0)	109 (35.8)	0	0
2009	669	13.9	96 (28.1)	96 (16.7)	20 (90.0)	20 (95.0)
2010	677	22.0	223 (30.4)	223 (20.6)	39 (92.3)	39 (74.4)
2011	669	15.8	27 (22.2)	27 (7.4)	28 (82.1)	28 (67.9)
2012	681	14.8	2 (100)	2 (50)	4 (75.0)	4 (50.0)
2013	683	7.9	3 (33.3)	3 (0)	4 (75.0)	4 (75.0)
2014	681	13.8	16 (31.3)	16 (25.0)	15 (100)	15 (66.7)
2015	380	17.1	4 (0)	4 (0)	3 (66.7)	3 (66.7)
2016	316	10.7	1 (0)	1 (0)	1 (100)	1 (0)

No. indicates the total number of children recruited each year. For 2007 and 2008, there were no corresponding first febrile samples stored due to operational constraints in those years.

### *ama1* and *mdr1* Deep Sequencing Data

The AmpSeq approach was validated using laboratory reference isolate (3D7 and Dd2) mixtures. For *ama1*, the 2 haplotypes representing both the laboratory controls were detected, whereas 3 laboratory haplotypes were detected for *mdr1* (Supplementary Figure 1). Overall, 57.8 million reads were obtained and following a series of quality control steps, a total of 13.8 million *ama1* and 2.96 million *mdr1* good-quality reads were generated from 484 samples (280 for *ama1* and 204 for *mdr1*) and 30 sequencing controls. The median read depth for *ama1* was 5658 reads (range, 4310–12 603) compared to 658 reads (range, 291–1676) for *mdr1*. Moreover, sequencing was less successful in asymptomatic infections, likely due to low parasitemia since samples that failed sequencing had a lower geometric mean (815 [range, 40–220 000] parasites/µL) compared to successfully sequenced samples (1474 [range, 40–600 482] parasites/µL). Similar observations were made with *mdr1* (Supplementary Table 2)

### Temporal Changes in *Pfmdr1* Drug Resistance Markers

**Figure 1.**
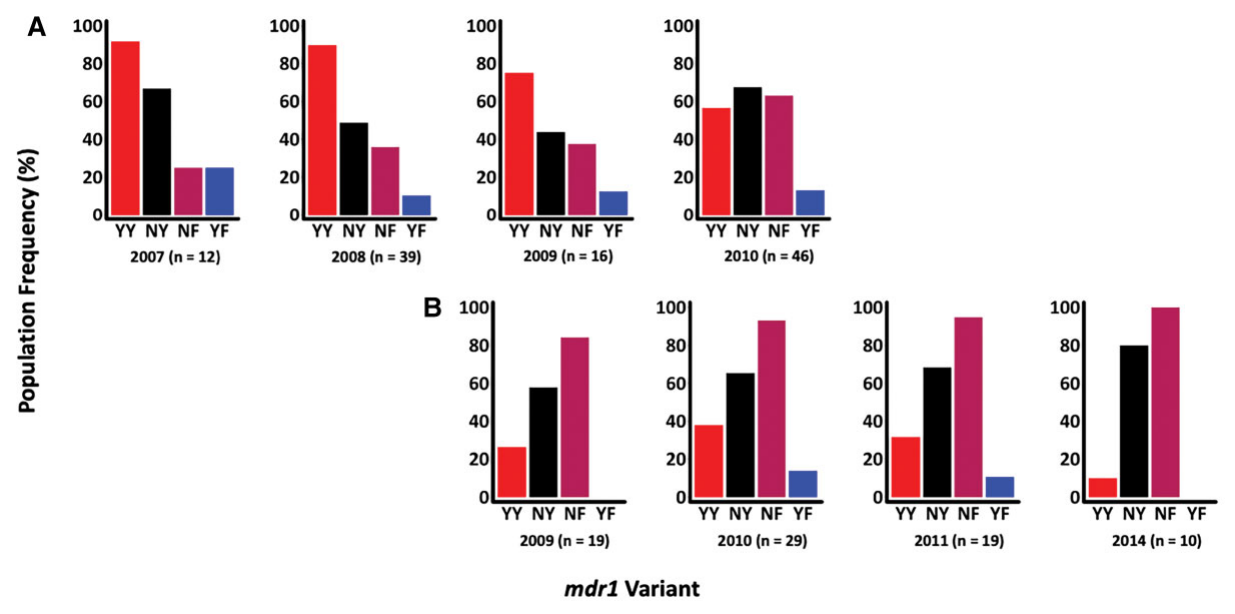
Population frequencies of *mdr1* haplotypes, based on codons 86 and 184, across time in asymptomatic (*A*) and febrile (*B*) infections. The x-axis shows the timepoints and haplotypes, and the y-axis represents the percentage population frequency. The *mdr1* population haplotype frequencies were calculated by counting the number of individuals harboring each haplotype at each time point. Each bar in the bar plots represents an *mdr1* haplotype as indicated by the labels below each bar. The n values represent the total number of samples in each time point. The 2012–2013 timepoints for febrile infections were dropped because n was <10.

Between 2007 and 2016, *mdr1* haplotypes were assessed across 120 and 84 successfully sequenced asymptomatic and first febrile samples, respectively. Four *mdr1* haplotypes were identified: NY (wild type) and YY, NF, and YF (mutant). There was a shift from YY in the years before 2009 to NY and NF post-2009 (Figure 1). However, the temporal increase in the frequencies of both the NY and NF haplotypes in asymptomatic and febrile infections was not statistically significant (NY: *P* = .7 and *P* = .4 and NF: *P* = .3 and *P* = .2, respectively). Likewise, the reduction in the frequency of the YY mutant haplotype over time in the 2 infections was also not significant (*P* = .6 and *P* = .8, respectively) (Figure 1). Notably, most participants harbored mixed *mdr1* haplotypes (60.2% and 75.3% in asymptomatic and febrile infections, respectively) with some participants harboring as many as 4 haplotypes (Supplementary Table 3)

### Temporal Changes in *Pfama1* Genetic Diversity and Complexity of Infection

**Figure 2.**
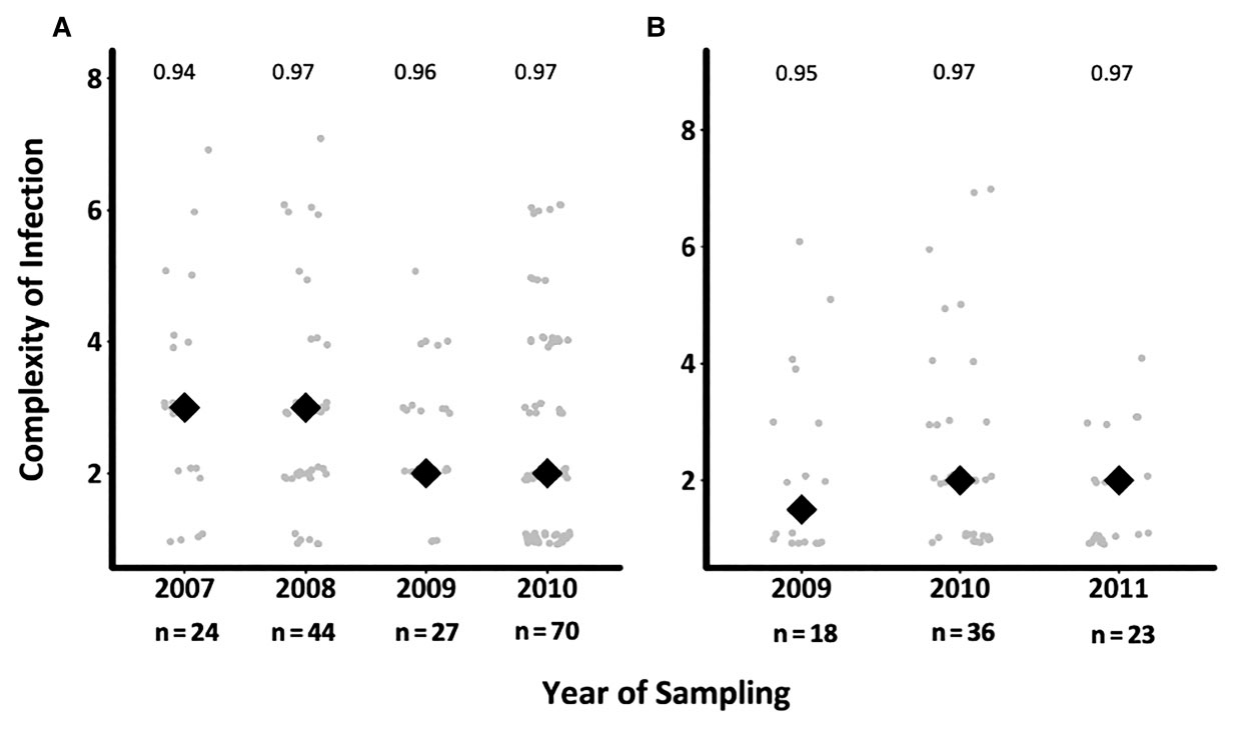
Temporal changes in complexity of infection (COI) across asymptomatic and first febrile infections. The figure shows the distribution of mean COI based on *ama1* over time in asymptomatic (*A*) and febrile (*B*) infections. The gray circles are the COI for the individuals genotyped, while the black diamonds are the mean COI per timepoint. The numbers above each time point represent the expected *ama1* heterozygosity. The graphs do not include samples beyond 2010 for asymptomatic infections and 2011 for febrile infections, since the samples were specifically selected as asymptomatic–febrile pairs, reducing the sample size to <10.

The genetic diversity of *ama1* was assessed across the 179 and 101 successfully sequenced samples in asymptomatic and first febrile infections, respectively. Of the 90 *ama1* haplotypes detected, numbered V1–V90, 79 and 62 haplotypes were observed in asymptomatic and first febrile infections, respectively, and the common haplotypes were evenly distributed over time (Supplementary Table 4). Rarefaction curves revealed that this study needed as many as 1000 samples at each time point to fully characterize *ama1* diversity (Supplementary Figure 2). Still, *ama1* revealed stable and high heterozygosity throughout the study period. A reduction in mean COI was observed across the years in the asymptomatic infections but was not significant (*P* = .4 and *P* = .18, respectively) (Figure 2). A higher mean COI was found in asymptomatic infections (mean, 2.73 [95% confidence interval [CI], 2.65–2.77) compared to febrile infections (mean, 2.22 [95% CI, 2.17–2.29]), and this was statistically significant (*P* = .01).

### Characteristics of Paired Asymptomatic and First Febrile Samples

A total of 30 and 17 children had paired asymptomatic and first febrile infection data for both *ama1* and *mdr1*, respectively. Among the 17 children with paired *mdr1* data, the NF and YF haplotypes increased from 32.3% to 45.5% and from 0 to 3.0%, respectively, whereas the NY and YY haplotypes decreased from 42.0% to 33.3% and from 25.8% to 18.2%, respectively (Supplementary Table 5). Three of the individuals (10%) had persistent *ama1* haplotypes, detected in the preceding asymptomatic infection. Eight individuals (26.7%) had a higher COI in their first febrile infection and an equal number of children had a lower COI, while the majority of children, 14 (46.7%), maintained their COI in both infections (Supplementary Table 5). Nine children with a lower COI during their first febrile infection were relatively older with a mean age of 8.1 years and had an increased time to their first febrile infection of 110.6 days, whereas 7 children with a higher COI during their first febrile infection were younger with a mean age of 6.9 years and had a shorter mean time to the first febrile infection of 97.4 days. About 17 children who maintained COI had a mean age of 6.3 years and an average time to the first febrile infection of 83 days. However, this analysis was underpowered due to a limited number of samples.

## DISCUSSION

AmpSeq revealed the extent of *ama1* and *mdr1* diversity in asymptomatic infections by improving the detection of minor haplotypes, the complexity of infections, and drug resistance genotypes.

The *mdr1*-YY haplotype is associated with decreased sensitivity to chloroquine and amodiaquine [27, 28], drugs that are no longer routinely used in Kenya, hence its temporal reduction. On the other hand, *mdr1*-NF is selected for by lumefantrine [28] and thus this is likely to be the main reason for its temporal increase. The *mdr1*-YF haplotype was initially detected as rare (<1%) [29] and was maintained at a stable frequency of approximately 10% from 2007 to 2011, indicating that it is more common than previously described. The impact of this haplotype on response to antimalarial treatment is not fully understood, though on a chloroquine background of the chloroquine resistance transporter (*crt*) CVIET haplotype, parasites transfected with the *mdr1*-YF haplotype had a high 50% inhibitory concentration to piperaquine, suggesting that *mdr1*-YF confers resistance to piperaquine [30]. The Kilifi population is 99% *crt*-CVMNK and thus the importance of YF in this population is yet to be explored, though it is unlikely to persist beyond 2014 since the Kilifi parasite population is predominantly >90% wild type at *mdr1* codon 86 in 2018 [31]. Many asymptomatic and febrile infections harbored mixed *mdr1* haplotypes (some as many as 4) that included the mutant genotypes. Thus, AmpSeq is capable of accurately detecting several haplotypes and highlights the great complexity of drug resistance genotypes per infection.

The identification of a greater COI in asymptomatic infections based on *ama1* AmpSeq compared to previous capillary sequencing is due to the higher sensitivity of deep-sequencing and its ability to detect minor haplotypes [32]. *The ama1* data revealed that the majority of asymptomatic infections were polyclonal (>60%), which was similar to proportions reported in malaria-endemic areas of western Kenya [33, 34] and Tanzania [35]. This high prevalence of polyclonal infections among asymptomatic infections may be the result of cumulative and frequent exposure to a large number of parasite haplotypes in malaria-endemic areas leading to the development of anti-disease immunity and asymptomatic infections as immunity to malaria is in part haplotype-specific [35, 36]. A previous study suggested that because febrile malaria infections tend to have higher parasite densities, sampling a greater number of parasites can increase the likelihood of detecting mixed infections [37]. In contrast, the ensuing first febrile infections in the current study were less polyclonal with a lower mean COI as they were the result of an infection with novel parasite haplotypes, probably unknown to the host protective immune responses, that outcompete and replace existing haplotypes [13]. The outcome was an increase in parasitemia in febrile infections to levels that were significantly higher than in asymptomatic infections (*t* test, *P* < .001), as revealed in this study, potentially leading to increased tissue damage and the manifestation of symptoms. However, we also note the limitation in our sampling frame to detect the full range of diversity using *ama1*, since the rarefaction analysis indicated the need for about 1000 samples per year. Still, only 3 *ama1* haplotypes were observed in the majority of time points, suggesting frequency-dependent selection due to variations in population immunity and hence the extensive number of circulating haplotypes.

Most of the paired asymptomatic febrile infections were due to a change in *ama1* haplotypes, and few individuals maintained the same haplotype profile during their first febrile infection. This is consistent with previous studies using the merozoite surface protein 2 (*msp2*) marker, suggesting that the parasites causing a clinical episode are those to which an individual has not yet mounted an efficient protective immune response [11–13, 37]. For the few persistent infections, it appears that only 1 individual who maintained the *ama1* haplotype at their first febrile infection is likely to have carried over the haplotype from the asymptomatic infection given the short, 10-day period between sampling. Conversely, the 2 individuals who maintained the dominant haplotypes in the population may have acquired these haplotypes from new infections since the time to reporting a first febrile episode was 96 and 155 days, respectively. To assess how long haplotypes are maintained in individuals, closer sampling frames are required between both infections. In contrast, individuals who had a reduction in haplotypes in their first febrile infection appeared to have a longer period between their asymptomatic and first febrile infections. This concurs with previous studies, which have reported that asymptomatic individuals with a higher COI were protected against febrile malaria [38], probably due to increased breadth of antibody responses [39]. This also agrees with the observation in this study where on average it was the older children who tended to have their first febrile infection with fewer haplotypes than they had during their asymptomatic infection.

This study had several limitations. The sequencing controls used with *ama1* and *mdr1* deep-sequencing permitted the detection of haplotypes only if they had a relative frequency of >5%, potentially missing haplotypes present below this threshold. Additionally, many samples were excluded since they had low parasite densities; hence, more than half of the total asymptomatic samples did not yield sequence data. However, a nested PCR approach may increase the PCR yield for samples with low parasitemia. Additionally, the sample size was small and may have confounded some of our findings, including the reduction of COI over time in asymptomatic infections and differences between asymptomatic and subsequent first febrile infections. Future work should increase the sample size to accurately determine these. The reliance on 1 polymorphic marker to characterize parasite diversity may have limited the detection of the full extent of parasite diversity per infection [32]. Nonetheless, the use of 1 highly polymorphic marker is appealing as including many markers complicates the detection of haplotypes and it is likely to be resource-intensive for use as a surveillance tool. The rate-limiting step, however, will be the generation of amplicons in duplicate and the concentration of each amplicon in the pooling strategy to ensure equal representation of each amplicon in the next generation sequencing reaction.

Notably, the temporal trends in both asymptomatic and febrile populations demonstrate the utility of AmpSeq in antimalarial drug resistance surveillance. Furthermore, AmpSeq provides a useful tool for tracking COI over time and identifying COI within and between infections to provide insights into the extent and the potential impact of interventions, such as drugs and vaccines, on parasite genetic diversity.

## Supplementary Data

Supplementary materials are available at *The Journal of Infectious Diseases* online (http://jid.oxfordjournals.org/). Supplementary materials consist of data provided by the author that are published to benefit the reader. The posted materials are not copyedited. The contents of all supplementary data are the sole responsibility of the authors. Questions or messages regarding errors should be addressed to the author.

## Supplementary Material

jiac144_Supplementary_Data

## Data Availability

The raw fastq files have been deposited in Zenodo (https://doi.org/10.5281/zenodo.6243929) under “(Fastq Files) Amplicon sequencing of ama1 and mdr1 to track within-host P. falciparum diversity in Kilifi, KENYA” (version 1) [dataset]. Data are available under the terms of the Creative Commons Attribution 4.0 International. The nucleotide sequence data reported in this paper are available in the GenBank database under the accession numbers *ama1* (MZ667649–MZ667738) and *mdr1* (MZ667739–MZ667747).

## Notes

***Acknowledgments.*** We thank the study participants for providing blood samples. Additionally, the following reagents were obtained through BEI Resources, National Institute of Allergy and Infectious Diseases, National Institutes of Health: Genomic DNA from *Plasmodium falciparum*, strain 3D7, MRA-102G (contributed by Daniel J. Carucci) and genomic DNA from *P. falciparum*, strain Dd2, MRA-150G (contributed by David Walliker).

***Disclaimer.*** The views expressed in this publication are those of the authors and not necessarily those of the African Academy of Sciences (AAS), the New Partnership for Africa’s Development Planning and Coordinating Agency (NEPAD), the Wellcome Trust, or the government of the United Kingdom.

***Financial support.*** This work was supported by the Developing Excellence in Leadership, Training and Science (DELTAS) Africa Initiative (grant number DEL-15-003) and a Wellcome Trust Intermediate Fellowship awarded to L. I. O.-O. (grant number 107568/Z/15/Z). The DELTAS Africa Initiative is an independent funding scheme of the AAS Alliance for Accelerating Excellence in Science in Africa and supported by NEPAD with funding from the Wellcome Trust (107769/Z/10/Z) and the government of the United Kingdom. J. A. B. and J. J. J. received support from the National Institutes of Health (grant numbers R01AI121558 and K24AI13499, respectively).
