# Supplementary material for: Amplicon Sequencing as a Potential Surveillance Tool for Complexity of Infection and Drug Resistance Markers in Plasmodium falciparum Asymptomatic Infections
Source: J Infect Dis. 2022 Apr 16;226(5):920–7. doi: 10.1093/infdis/jiac144 (PMC7613600; doi:10.1093/infdis/jiac144)
Supplement: jiac144_Supplementary_Data [file jiac144_supplementary_data.pdf]

Supplementary Material

Supplementary Table 1. List of primers used for PCR amplification prior to deep sequencing.

Gene (GeneID)	Primer	Sequence '5-'3 [MID-Primer]	Modification	Length in bases
ama1 (PF3D7_1133400)	AMA1_F01	**ACGAGTGCGT**GAAATGTCCAGTATTTGGTAAAGG	None	34
	AMA1_F02	**ACGCTCGACA**GAAATGTCCAGTATTTGGTAAAGG	None	
	AMA1_F03	**AGACGCACTC**GAAATGTCCAGTATTTGGTAAAGG	None	
	AMA1_F04	**AGCACTGTAG**GAAATGTCCAGTATTTGGTAAAGG	None	
	AMA1_F05	**ATCAGACACG**GAAATGTCCAGTATTTGGTAAAGG	None	
	AMA1_F06	**ATATCGCGAG**GAAATGTCCAGTATTTGGTAAAGG	None	
	AMA1_F07	**CGTGTCTCTA**GAAATGTCCAGTATTTGGTAAAGG	None	
	AMA1_F08	**CTCGCGTGTC**GAAATGTCCAGTATTTGGTAAAGG	None	
	AMA1_F09	**TCTCTATGCG**GAAATGTCCAGTATTTGGTAAAGG	None	
	AMA1_F10	**TGATACGTCT**GAAATGTCCAGTATTTGGTAAAGG	None	
	AMA1_F11	**CATAGTAGTG**GAAATGTCCAGTATTTGGTAAAGG	None	
	AMA1_F12	**CGAGAGATAC**GAAATGTCCAGTATTTGGTAAAGG	None	
	AMA1_F13	**ATACGACGTA**GAAATGTCCAGTATTTGGTAAAGG	None	
	AMA1_F14	**TCACGTACTA**GAAATGTCCAGTATTTGGTAAAGG	None	
	AMA1_F15	**CGTCTAGTAC**GAAATGTCCAGTATTTGGTAAAGG	None	
	AMA1_F16	**TCTACGTAGC**GAAATGTCCAGTATTTGGTAAAGG	None	
	AMA1_F17	**TGTACTACTC**GAAATGTCCAGTATTTGGTAAAGG	None	
	AMA1_F18	**ACGACTACAG**GAAATGTCCAGTATTTGGTAAAGG	None	
	AMA1_F19	**CGTAGACTAG**GAAATGTCCAGTATTTGGTAAAGG	None	
	AMA1_F20	**TACGAGTATG**GAAATGTCCAGTATTTGGTAAAGG	None	
	AMA1_F21	**TACTCTCGTG**GAAATGTCCAGTATTTGGTAAAGG	None	
	AMA1_F22	**TAGAGACGAG**GAAATGTCCAGTATTTGGTAAAGG	None	
	AMA1_F23	**TCGTCGCTCG**GAAATGTCCAGTATTTGGTAAAGG	None	
	AMA1_F24	**ACATACGCGT**GAAATGTCCAGTATTTGGTAAAGG	None	
	AMA1_F25	**ACGCGAGTAT**GAAATGTCCAGTATTTGGTAAAGG	None	
	AMA1_F26	**ACTACTATGT**GAAATGTCCAGTATTTGGTAAAGG	None	
	AMA1_R	CCCATAATCCGAATTTTGCATTC	None	23
mdr1 (PF3D7_0523000)	MDR1_F01	**ACGAGTGCGT**TGTATGTGCTGTATTATCAGGAGG	None	34
	MDR1_F02	**ACGCTCGACA**TGTATGTGCTGTATTATCAGGAGG	None	
	MDR1_F03	**AGACGCACTC**TGTATGTGCTGTATTATCAGGAGG	None	
	MDR1_F04	**AGCACTGTAG**TGTATGTGCTGTATTATCAGGAGG	None	
	MDR1_F05	**ATCAGACACG**TGTATGTGCTGTATTATCAGGAGG	None	
	MDR1_F06	**ATATCGCGAG**TGTATGTGCTGTATTATCAGGAGG	None	
	MDR1_F07	**CGTGTCTCTA**TGTATGTGCTGTATTATCAGGAGG	None	
	MDR1_F08	**CTCGCGTGTC**TGTATGTGCTGTATTATCAGGAGG	None	
	MDR1_F09	**TCTCTATGCG**TGTATGTGCTGTATTATCAGGAGG	None	
	MDR1_F10	**TGATACGTCT**TGTATGTGCTGTATTATCAGGAGG	None	
	MDR1_F11	**CATAGTAGTG**TGTATGTGCTGTATTATCAGGAGG	None	
	MDR1_F12	**CGAGAGATAC**TGTATGTGCTGTATTATCAGGAGG	None	
	MDR1_F13	**ATACGACGTA**TGTATGTGCTGTATTATCAGGAGG	None	
	MDR1_F14	**TCACGTACTA**TGTATGTGCTGTATTATCAGGAGG	None	
	MDR1_F15	**CGTCTAGTAC**TGTATGTGCTGTATTATCAGGAGG	None	
	MDR1_F16	**TCTACGTAGC**TGTATGTGCTGTATTATCAGGAGG	None	
	MDR1_F17	**TGTACTACTC**TGTATGTGCTGTATTATCAGGAGG	None	
	MDR1_F18	**ACGACTACAG**TGTATGTGCTGTATTATCAGGAGG	None	

MDR1_F19	**CGTAGACTAG**TGTATGTGCTGTATTATCAGGAGG	None	
MDR1_F20	**TACGAGTATG**TGTATGTGCTGTATTATCAGGAGG	None	
MDR1_F21	**TACTCTCGTG**TGTATGTGCTGTATTATCAGGAGG	None	
MDR1_F22	**TAGAGACGAG**TGTATGTGCTGTATTATCAGGAGG	None	
MDR1_F23	**TCGTCGCTCG**TGTATGTGCTGTATTATCAGGAGG	None	
MDR1_F24	**ACATACGCGT**TGTATGTGCTGTATTATCAGGAGG	None	
MDR1_F25	**ACGCGAGTAT**TGTATGTGCTGTATTATCAGGAGG	None	
MDR1_F26	**ACTACTATGT**TGTATGTGCTGTATTATCAGGAGG	None	
MDR1_R	CCCATTAAAGCCTCTTCTATAATGG	None	25

MID tags are shown in bold face while the forward primers are shown in regular faces.

Supplementary Table 2. A comparison of parasitaemia between samples that were successfully sequenced compared to those that failed

Marker	Sample	Sequencing Success	*n*	Parasitemia Per Microlitre		
				Min	Geometric Mean	Max
ama1	Asymptomatic	failed	356	40	815.0	220,000
		success	179	40	1,474.0	600,482
	Febrile	failed	13	2,801	27,556.6	510,001
		success	101	2,561	47,050.1	1,280,001
mdr1	Asymptomatic	failed	415	40	56.0	156,214
		success	120	46	1,207.0	594,119
	Febrile	failed	30	2,738	34,702.0	510,001
		success	84	2,274	42,004.1	1,280,007

This table summarizes the mean, geometric mean and maximum parasitaemia (per microlitre) as determined by microscopy. Additionally, samples are stratified by markers (*ama1*) and infection (asymptomatic and febrile) . Across all markers, there was a higher geometric mean parasitaemia in samples that were successfully genotyped compared to those that failed.

Supplementary Table 3. Temporal distribution of *mdr1* haplotypes in asymptomatic and febrile infections.

Infection	Haplotypes	2007 [n=12]	2008 [n=39]	2009 [n=35]	2010 [n=75]	2011 [n=19]	2014 [n=10]
Asymptomatic				Monoclonal infection, n [%]			
	NY	1 [8.3]	1 [2.6]	2 [12.5]	7 [15.2]		
	NF			2 [12.5]	6 [13]		
	YY	4 [33.3]	16 [41]	5 [31.2]	3 [6.5]		
				Mixed infection, n [%]			
	NF/NY/YF/YY	3 [25]	3 [7.7]	1 [6.2]	4 [8.7]		

	NY/YY	4 [33.3]	7 [17.9]	3 [18.8]	6 [13]		
	NF/NY		3 [7.7]		7 [15.2]		
	NF/NY/YY		6 [15.4]	1 [6.2]	9 [19.6]		
	NF/YY		2 [5.1]	1 [6.2]	2 [4.3]		
	YF/YY		1 [2.6]		1 [2.2]		
	NF/YF/YY			1 [6.2]	1 [2.2]		
**Febrile**	**Monoclonal infection, n [%]**						
	NY			1 [5.3]	1 [3.4]	1 [5.3]	
	NF			5 [26.3]	6 [20.7]	3 [15.8]	1 [10]
	YY			1 [5.3]			
	**Mixed infection, n [%]**						
	NF/NY			8 [42.1]	11 [37.9]	9 [47.4]	8 [80]
	NF/YY			1 [5.3]	3 [10.3]	2 [10.5]	1 [10]
	NY/YY			1 [5.3]	1 [3.4]		
	NF/NY/YY			2 [10.5]	3 [10.3]	2 [10.5]	
	NF/NY/YF/YY				4 [13.8]	2 [10.5]	

Individuals harbouring only one *mdr1* haplotype were classified as monoclonal infection while those harbouring more than one haplotype were classified as a mixed infection. The relative frequencies were calculated by counting the number of individuals harbouring the respective haplotypes per infection per timepoint. n represents the total number of individuals per timepoint.

Supplementary Table 4. Relative *ama1* haplotype frequencies across asymptomatic and febrile infections.

Haplotype ID	Haplotype haplotype	Population n [%]	Relative Frequency [%]							
			Asymptomatics, Year [n]				Febrile, Year [n]			
			2007 [24]	2008 [44]	2009 [27]	2010 [70]	2009 [18]	2010 [36]	2011 [27]	2014 [21]
V1	KTENDNPMNGRDLKNEYMNDNEDKDKEKKSQNDEE	71 [24.2]	29.3	16.5	11.2	6.5	5.5	7.3	2.6	8.3
V2*	NTENDKLMDQRHFKDKYMIDNKDKDKEKISQNDEK	54 [18.4]	0.5	4.4	14.9	4.6	27.1	9.6	6.6	7.0
V3	NKGNDELIDDRDFKNEYMNDNQYEYKQKISQNDEK	41 [14]	3.7	4.5	6.5	8.2	18.9	10.9	-	0.1
V4	NTGNYKLMDDRLLKDEDMNDNKYNDKEKKSQNDEE	16 [5.5]	1.1	2.2	7.3	0.3	-	-	-	-
V5*	NTGNYELMDERHFKDKYMIDNKDKDKEKISQNDEK	1 [0.3]	-	-	-	1.4	-	-	-	-
V6	NTENDNLINGKDFKDEDMNDKEYEDNEKKSQNDEK	33 [11.3]	8.9	3.7	4.9	1.0	-	8.2	4.3	12.9
V7	NTENDELMDRRDFKNEDMNDKEYKDKEKKSQNDEK	32 [10.9]	4.8	9.2	-	5.3	-	0.4	-	0.3
V8*	NTGKDNLINGRDLKNEDMNDNKDKNKQKKSQNDEK	30 [10.2]	1.6	4.1	2.7	1.2	0.3	6.0	4.4	0.1
V9	NTENDKLMDQRHFKDKYMIDNKDKDKQIISQNDEK	25 [8.5]	0.9	4.2	4.4	5.0	0.2	1.4	6.9	-
V10	KTENDELMDDRRFKDEYMNDNKYNDKEKKSQNDEK	25 [8.5]	2.7	0.5	2.7	5.3	3.7	3.3	3.9	6.7
V11	NTENDKHMDDRLLKDEDMIDNKDKDKQKKSQDDEK	22 [7.5]	4.3	1.1	0.3	1.2	5.7	2.4	11.0	7.1
V12	NTENDKLMDQRHFKDEDMIDNKDKDKEKKLQNHEE	19 [6.5]	2.3	3.0	2.6	-	1.9	2.8	1.7	6.2
V13	KTENDNPMNGRDLKNEYMNDNKYNDNEKKSQNDEK	14 [4.8]	-	3.7	2.5	4.3	-	0.9	-	4.2
V14	NKGNDNLINGRDFKNEYMNDNKYNDKQKKLENDEE	13 [4.4]	0.7	1.6	-	0.4	-	3.0	10.1	-
V15	KTENDNPMNGRDLKNEYMNDNEDKDKEKKSQNDEK	13 [4.4]	2.9	1.1	-	1.6	-	0.1	-	-
V16	NTGKDNLINGRDLKNEDMNDNKYNDKQKKLENDEE	12 [4.1]	-	3.3	5.1	3.2	-	4.7	-	-
V17	KTENDNPMNGRDLKNEYMNDNKYNDNEKKSQNDEE	12 [4.1]	-	1.6	-	1.7	-	2.6	3.3	0.8
V18	NTENDELMDQRHFKDKYMIDNKDKDKQIISQNDEK	10 [3.4]	-	-	3.1	1.8	-	0.2	3.3	-
V19*	NTGKDNLINGKDFKDEDMNDKEYKDKEKKSQNDEK	11 [3.8]	-	1.3	8.3	0.9	-	0.4	-	4.1
V20	KTENDELIDQRHLKDEYMNDNKYEDKEKKLENDEE	9 [3.1]	4.4	-	0.1	2.8	-	2.7	-	6.3
V21 (V19)	NTGKDNLINGKDFKDEDMNDKEYKDKEKKSQNDEK	9 [3.1]	-	1.1	-	1.4	-	1.6	-	-
V22	NTENYELMDERHFKDKYMIDNKDKDKEKISQNDEK	9 [3.1]	-	0.6	0.6	3.8	0.8	-	0.1	-
V23	NKGNDELINGRDFKNEYMNDKEDKDKEKKSQNDEK	9 [3.1]	-	2.3	-	0.2	2.7	2.8	0.2	2.6
V24	NTGNYEHMDERHFKDKYMIDNKYEDKQKKLQNDEE	9 [3.1]	1.8	0.1	-	2.3	0.6	0.2	-	-
V25	NKGNDELIDDRDFKNEYMNDNQYEDKQKKSQNHEK	8 [2.7]	1.7	2.9	-	0.8	-	-	-	-
V26	NKGNDKLIDDRDFKNEYMNDKEYKDKEKKSQNHEK	8 [2.7]	-	0.7	-	1.5	-	0.5	4.4	-
V27	NKGNDNLINGRDFKNEYMNDNKYEDKQKKLQNHEE	8 [2.7]	-	0.2	1.8	0.8	-	0.1	0.4	-
V28	KTENDKLMDQRHFKDKYIIDNKDKDKEKISQNDEE	7 [2.4]	-	-	-	3.7	3.0	0.2	-	4.5
V29	KTENDELMDDRRFKDEYMNDNKYNDKEKKLENDEE	7 [2.4]	-	0.2	-	1.9	4.5	2.2	-	-

V30	NTENDNLINGRDLKNEDMNDNKDKNKQKKSQNDEK	7 [2.4]	3.3	0.6	1.9	0.5	-	2.2	-	-
V31	NTENDKLIDQRDLKNEYMNDNEYKDKQKKLENDEK	7 [2.4]	-	-	-	-	0.2	0.4	4.4	6.8
V32	NKGNDNLINGRDFKNEYMNDNEDKDKEKKSQNDEK	7 [2.4]	-	1.6	1.1	0.4	-	0.3	-	0.4
V33	NTGKDNLINGRDLKNEDMNDNKDKNKQKKLQNDEE	6 [2]	6.8	0.2	-	-	-	-	0.0	-
V34	NTENYELMDERHFKDKYMIDNKDKDKEKKSQNDEE	6 [2]	-	0.6	2.9	0.1	3.0	-	4.1	-
V35	NTENDKLMDERHFKDKYMIDNKDKDKEKISQNDEK	6 [2]	-	3.9	-	-	-	-	0.5	-
V36 (V8)	NTGKDNLINGRDLKNEDMNDNKDKNKQKKSQNDEK	6 [2]	-	0.3	1.3	1.1	-	-	4.3	0.8
V37	NTENDKLMDQRDFKNEYMNDNEYKDKQKKLENHEK	6 [2]	-	1.1	0.3	1.4	-	0.1	-	-
V38	NTENDNLINGKDFKDEDMNDKEYEDNEKKSQNDEE	6 [2]	-	-	0.1	0.8	2.0	-	0.0	-
V39	NKGNDNLINGRDFKNEYMNDNKYNDKQKKLQNHEE	5 [1.7]	8.8	-	-	-	-	-	-	-
V40	KTENDELIDDRDSKDEYMNDNEYKDKEKKSQNDEK	5 [1.7]	-	1.5	0.4	0.1	-	2.8	-	-
V41	NKGNDELIDDRDFKNEYMNDNQYEDKQKKLQNDEE	5 [1.7]	-	0.1	3.2	0.1	-	-	-	-
V42	KTENDNPMNGRDLKNEYMIDNKDKDKEKKLENDEE	5 [1.7]	-	-	0.1	0.3	0.1	0.4	-	-
V44	KTENDELMDDRRFKDEYMNDNKYNDNEKKSQNDEK	4 [1.4]	-	-	-	2.6	5.6	-	-	-
V45	NTGNYELMNGRDLKNEDMNDNKDKNKQKKSQNDEK	4 [1.4]	-	-	-	1.0	-	1.2	4.3	-
V46	NTENDELMDRRDFKNEDMNDNKDKNKQKKSQNDEE	4 [1.4]	-	1.3	-	1.0	-	-	-	-
V47	NKGNDKLIDDRDFKNEYMNDNQYEDKQKKLQNHEK	4 [1.4]	-	-	0.1	1.9	-	2.6	-	-
V48	KTENDNPMNGRDLKNEDMNDNKDKNKQKKSQNDEK	4 [1.4]	-	-	-	1.4	-	-	-	8.2
V49	NKGNDELIDQRHFKDEYMNDNKYEDKQKKSQNDEK	4 [1.4]	-	-	0.7	1.6	-	-	-	-
V50	NKGNDELIDDRDFKDEDMNDKEYKDKEKKSQNDEK	4 [1.4]	0.8	-	0.3	0.4	-	1.4	-	-
V51	NKGNDNPMNGRDLKNEYMNDNQDKDKEKKSQNDEE	4 [1.4]	-	2.2	-	-	-	0.0	-	-
V52	NTENDNPMNGRDLKNEYMNDNEDKDKEKKSQNDEE	4 [1.4]	0.5	-	-	-	-	2.8	-	-
V53	NTENDNLIDHRDSKNEYMNDNQYKDKQKKSQNDEK	3 [1]	-	0.3	-	2.3	-	-	-	-
V54 (V5)	NTGNYELMDERHFKDKYMIDNKDKDKEKISQNDEK	3 [1]	-	2.8	-	-	-	-	-	-
V55	NTGNYEHMDERHFKDKYMIDNKYEDKQKKLQNDEK	3 [1]	0.5	-	-	0.1	-	2.8	-	-
V56	KTENDKLMDDRDLKNEYMNDNKYNDNEKKSQNDEK	3 [1]	-	0.3	-	1.3	-	0.2	-	-
V57	NKGNDELIDDRDFKNEYMNDNQYEDKQIISQNDEE	3 [1]	-	-	3.6	0.1	-	-	-	-
V58	NTGNYEHMDERHFKDKYMIDNKYEDKQIISQNDEK	3 [1]	4.2	-	-	-	-	-	4.4	-
V59	NTENDNLINGKDFKDEDMNDKEYKDKEKKSQNDEK	2 [0.7]	-	-	-	0.7	-	-	-	-
V60	KTENDKLMDRRHLEDEYMNDNEYKDKEKKLENDEK	2 [0.7]	-	-	-	0.5	-	2.5	-	-
V61	NTENDKHMDDRVLKDEYMNDNEDKDKQKKSQNDEK	2 [0.7]	-	-	-	1.4	-	-	-	-
V62	NTENDNPMDQRHFKDKYMIDNKDKDKEKKLENDEK	1 [0.3]	-	0.3	-	-	-	-	-	-
V63	NTGKDNLINGKDFKDEDMNDKEYEDNEKKSQNDEK	1 [0.3]	-	-	-	1.5	-	-	-	-
V64	NTENDKLIDQRDLKNEYMNDNKYNDKQKKLENDEE	2 [0.7]	-	2.1	-	-	-	0.2	-	-
V65	NKGNDNLMNGRDFKNEYMNDNKYEDKQKKLQNHEE	2 [0.7]	-	0.0	-	-	5.6	-	-	-

V66	NKGNDNLMNGRDFKNEYMNDNKYNDKEKKSQNDEK	2 [0.7]	-	-	2.5	0.1	-	-	-	-
V67	NTENDKLIDQRDLKNEYMNDNEYKDKQKKLENHEK	2 [0.7]	-	1.7	-	-	-	-	-	-
V68	NKGNDELIDQRHLKDEYMNDNKYEDKEKKLENDEE	2 [0.7]	-	0.0	-	-	3.0	-	-	-
V69	NTVNDKLMDDRLLKDEDMIDNKDKDKEKKLENDEK	2 [0.7]	-	1.0	-	0.1	-	-	-	-
V70	NKGNDELIDDRDFKNEYMNDNKYEDKQKKLQNHEK	2 [0.7]	0.9	-	-	-	-	-	-	-
V71	NTGNYKLMDDRLLKDEDMIDNKDKDKEKKLENDEK	2 [0.7]	-	-	-	1.4	-	-	-	-
V72 (V2)	NTENDKLMDQRHFKDKYMIDNKDKDKEKISQNDEK	2 [0.7]	-	2.2	-	-	-	-	-	-
V73	NKGNDELIDQRHFKDEYMNDNKYEDKQKKLQNHKK	2 [0.7]	-	-	2.8	-	-	-	-	-
V74	NTGNYKLMDDRLLKDEDMIYNKDKDKQKKLENDEK	1 [0.3]	2.9	-	-	-	-	-	-	-
V75	NTENDKLMDQRHFKDKYIIDNKDKDKEKISQNDEK	1 [0.3]	-	-	-	1.0	-	-	-	-
V76	NTENDKLIDQRDLKNEYMNDNKYNDKEKKSQNDEK	1 [0.3]	-	-	-	0.9	-	-	-	-
V77	NTGNYEHMDERHFKDKYMIDNKDKDKQIISQNDEK	1 [0.3]	-	0.9	-	-	-	-	-	-
V78	NTGKDELIDDRDFKNEYMNDNQYEDKQKKSQNHEK	1 [0.3]	-	0.8	-	-	-	-	-	-
V79	KTENDELIDQRHLKDEYMNDNQYEYKEKKLENDEE	1 [0.3]	-	-	-	0.4	-	-	-	-
V80	NTGKDDLINGRDLKNEDMNDNKDKNKQKKLQNDEE	1 [0.3]	-	-	-	0.4	-	-	-	-
V81	NTGNDNLMNGRDFKNEYMNDNEDKDKEKKSQNDEK	1 [0.3]	-	-	-	-	-	3.3	-	-
V82	KTENDNLMDQRHFKDKYMIDNKDKDKEKKLENDEE	1 [0.3]	-	-	-	-	5.9	-	-	-
V83	KTENDNPMDDRLLKDEDMNDNKDKDKEKKSQNDEK	1 [0.3]	-	-	-	-	-	0.0	4.4	-
V84	KTENDELINGKDFKDEDMNDKEYKDKEKKSQNDEK	1 [0.3]	-	-	-	-	-	2.8	-	-
V85	NTENDKLMDQRHFKDEDMIDNKDKDKQKISQNDEK	1 [0.3]	-	-	-	-	-	-	4.4	-
V86	NTGKDNLINGKDFKDEDMNDKEYKDKQKKSQNHEK	1 [0.3]	-	-	-	-	-	-	4.4	-
V87	KTENDKLMDQRHFKDKYMIDNKDKDKEKISQNDEK	1 [0.3]	-	-	-	-	-	-	-	6.4
V88	NKGNDELIDQRHFKDEYMNDNKYEDKQIKLQNHEK	1 [0.3]	-	-	-	-	-	-	-	6.4
V89	NKGNDELIDDRDFKNEYMNDKEYKDKQKKLQNDEE	1 [0.3]	-	-	-	-	-	-	-	-
V90	NTENDKLIDQRDLKNEYMNDNKYNDKEKKSQNDEE	1 [0.3]	-	-	-	-	-	-	2.0	-

The translated DNA sequences for ama1 span codons 156-307 of the full ama1 protein and shown here are the amino acid haplotypes including the following 35 codons 162, 167, 172, 173, 175, 187, 189, 190, 196, 197, 199, 200, 201, 203, 204, 206, 207, 224, 225, 227, 228, 230, 242, 243, 244, 245, 267, 269, 282, 283, 285, 286, 296, 299 and 300. Some haplotypes (marked with *) varied at the DNA level but collapsed into identical amino acid haplotypes includin: V2 and V72 (synonymous SNP codon 308), V4 and V54 (synonymous SNP codon 292), V8 and V36 (synonymous SNP codon 308) as well V19 and V21 (synonymous SNP codon 308). Entries marked with “-” represent haplotypes that were not detected in the respective infections.

Supplementary Table 5. A comparison of paired asymptomatic and first-febrile infections for *ama1* and *mdr1* markers.

Study Number	Year	Age	Days to Fever	Identity by *ama1*	***AMA1*** Asymptomatic						***AMA1*** First-Febrile				***MDR1*** Asymptomatic			***MDR1*** First-Febrile			
PID_01	2009	7.9	96	Persistent	**V2**						**V2**	V34	V11								
PID_02	2008	8.8	300	Novel	V8	V6					V2				YY			NY	NF		
PID_03	2015	5.3	155	Persistent	**V1**	V6	V2				**V1**	V11	V2	V48	NY	YY		NF			
PID_04	2008	5.1	326	Novel	V41	V9	V32	V3			V2										
PID_05	2008	8.6	102	Novel	V6	V19					V42	V31	V11	V10							
PID_07	2009	7.2	37	Novel	V8	V4					V10	V3			NY	YY		YY			
PID_11	2009	6.9	42	Novel	V36	V6					V65				YY			NF			
PID_12	2010	4.8	227	*ND*											NF			NF	NY		
PID_13	2010	5.7	10	Persistent	V25	V14	**V18**	V11	V3	V19	V51	V17	**V18**	V1							
PID_14	2010	9.5	77	Novel	V37	V5					V6	V3									
PID_15	2010	7.4	46	Novel	V37						V53										
PID_16	2010	5.3	106	Novel	V26						V3	V2					NF	NF	NY		
PID_18	2010	9.3	216	Novel	V28						V50	V3	V8		NY			NF	YY		
PID_19	2010	7.3	21	Novel	V3	V28	V58	V18	V29	V90	V14	V68			NY	NF	YY	NF	YY		
PID_20	2010	9.1	15	Novel	V16									V8							
PID_21	2010	3	55	Novel	V7						V28	V8	V3	V29							
PID_22	2010	2.7	23	*ND*													NF	NF			
PID_23	2010	7.4	86	Novel	V23	V10	V15	V13	V2		V16				NF	NY	YY	NF	NY		
PID_25	2009	9.6	69	Novel	V2	V3	V38	V1	V7	V13	V12				NF	YY		NF	YY	NY	YF
PID_22	2010	5.7	23	Novel	V3						V27	V20									
PID_24	2010	8.8	53	Novel	V64						V42	V1	V50								
PID_27	2011	9.9	35	Novel	V33						V27	V11	V12								
PID_29	2010	5.7	23	*ND*											NY	NF					NY
PID_30	2011	8.4	63	Novel	V10	V18	V30				V2	V43			NY	NF	NY	NY	NF	YY	
PID_32	2011	7	115	Novel	V6	V1								V26							
PID_33	2011	3.6	25	Novel	V1						V23	V2	V14	V9							
PID_35	2012	8.6	203	Novel	V60						V43	V35	V17								
PID_36	2012	12	289	Novel	V17	V25	V2				V53				YY	NY	NF	YY	NY	NF	
PID_37	2009	7.1	40	Novel	V48	V8					V11	V20			NY			NY	NF		
PID_39	2011	10.3	60	Novel	V11						V2	V36	V12		NY			NY	NF		
PID_42	2011	1.9	36	Novel	V21						V31	V14									
PID_44	2014	1.9	22	Novel	V18						V6										
PID_45	2010	7.5	92	Novel	V12						V79	V7			NY	NF	NY	NY	NF		

The persistent haplotypes are in bold and shaded grey. The change in frequencies of these persistent haplotypes between the asymptomatic and first-febrile infections are as follows: V2 (100 to 44.1%), V1 (48.3 to 63.9%) and V18 (0.7 to 6.2%). *ND* represents infections that whose identity could not be determined since *ama1* data was missing.

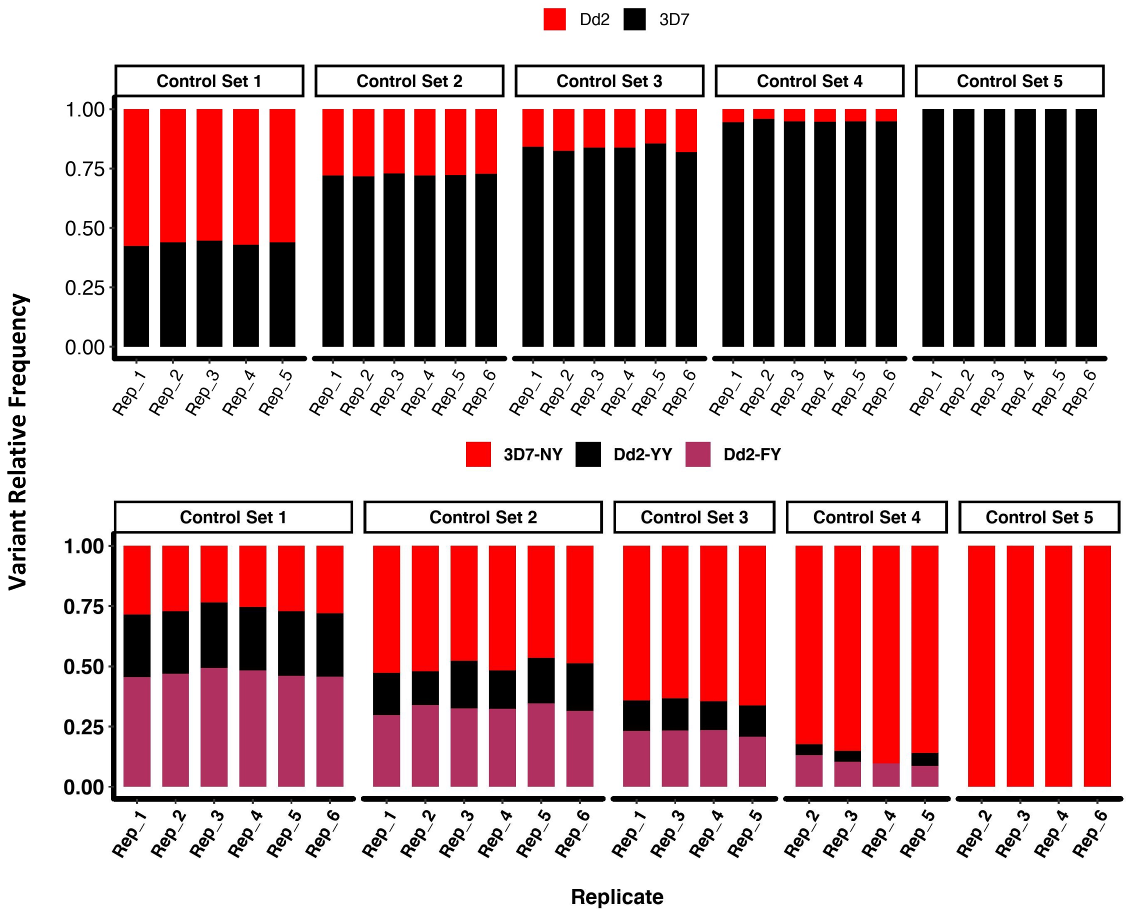

Supplementary Figure 1. Detection of *ama1* (top panel) and *mdr1* (bottom panel) haplotypes in laboratory reference isolate controls. These deep sequencing controls were prepared by mixing the laboratory isolates (3D7 and Dd2) as six replicates each in the following proportions: 100%:100%, 75%:25%, 85%:15%, 95%:5% and 100%:0%, respectively. Two *ama1* haplotypes (3D7 and Dd2) were expected and three *mdr1* haplotypes (3D7-NY, Dd2-YY and Dd2-YF). *ama1* sequencing failed for replicate 6 of Control Set 1, while for *mdr1,* sequencing failed for replicates 2 and 6 of control set 3, replicates 1 and 6 of control set 4 and replicates 1 and 5 of control set 5. The 3D7 and Dd2 haplotypes were detected in proportions similar to what was expected based on the input ratios.

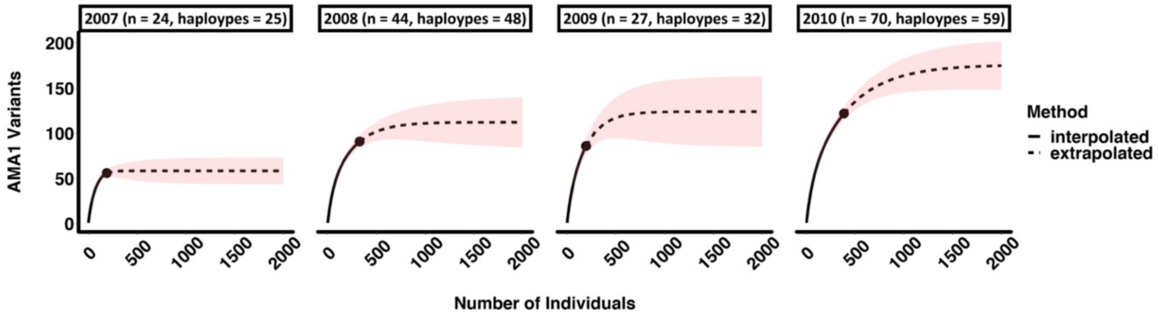

Supplementary Figure 2. ***ama1*** **rarefaction curves.** Using a sample size of 2,000 as the endpoint for rarefaction extrapolation, an estimate of *ama1* haplotype richness was conducted for timepoints that had more than 10 samples (2007-2010). Based on plateauing of the curves, the greatest under-sampling for ama1 was detected in 2010. It appears this study would have needed to sample as many as 1000 infections per timepoint to fully characterize the diversity of *ama1*.
